# Functional MRI Reveals Locomotion-Control Neural Circuits in Human Brainstem

**DOI:** 10.3390/brainsci10100757

**Published:** 2020-10-20

**Authors:** Pengxu Wei, Tong Zou, Zeping Lv, Yubo Fan

**Affiliations:** 1 School of Biological Science and Medical Engineering and Beijing Advanced Innovation Center for Biomedical Engineering, Beihang University, 37# Xueyuan Road, Haidian District, Beijing 100083, China; pengxuwei@buaa.edu.cn (P.W.); zonghekangfuke@med.nrcrta.cn (Z.L.); 2 Beijing Key Laboratory of Rehabilitation Technical Aids for Old-Age Disability, Key Laboratory of Neuro-Functional Information and Rehabilitation Engineering of the Ministry of Civil Affairs, National Research Center for Rehabilitation Technical Aids, No. 1 Ronghuazhong Road, Beijing Economic and Technological Development Zone, Beijing 100176, China; snowing9061@126.com

**Keywords:** locomotion, human brainstem, cuneiform nucleus, pedunculopontine nucleus, functional magnetic resonance imaging

## Abstract

The cuneiform nucleus (CN) and the pedunculopontine nucleus (PPN) in the midbrain control coordinated locomotion in vertebrates, but whether similar mechanisms exist in humans remain to be elucidated. Using functional magnetic resonance imaging, we found that simulated gait evoked activations in the CN, PPN, and other brainstem regions in humans. Brain networks were constructed for each condition using functional connectivity. Bilateral CN–PPN and the four pons–medulla regions constituted two separate modules under all motor conditions, presenting two brainstem functional units for locomotion control. Outside- and inside-brainstem nodes were connected more densely although the links between the two groups were sparse. Functional connectivity and network analysis revealed the role of brainstem circuits in dual-task walking and walking automaticity. Together, our findings indicate that the CN, PPN, and other brainstem regions participate in locomotion control in humans.

## 1. Introduction

Locomotion is a fundamental ability in humans. Gait performance is related to the risk of dementia [1] and falls in the elderly [2] that lead to heavy burdens, and can be damaged by critical brain illnesses such as Parkinson’s disease [3] and stroke [4]. In the mesencephalic locomotor region (MLR) of vertebrates, the cuneiform nucleus (CN) supports defensive forms of locomotion, such as high-speed running to escape from dangerous contexts, whereas the pedunculopontine nucleus (PPN) may mediate slow, exploratory locomotion [5,6]. Descending dopaminergic projections from the substantia nigra to MLR possibly play a role in the locomotor deficits in Parkinson’s disease [7]. Stimulating the MLR improves paretic hindlimb function in mice [8], indicating a therapeutic potential [9].

The existence of MLR in non-human primates has been proved [10]. However, the role of the MLR in locomotion of humans remains to be elucidated. Although mental imagery of walking or running in healthy subjects was found to activate the brainstem, the findings cannot exactly predict responses of the human brainstem evoked by real-time bipedal movements detected with functional magnetic resonance imaging (fMRI) [11,12,13].

Various technical and methodological challenges limit the application of brainstem fMRI [14]. Recent advances including the brainstem co-registration [15] and analysis using brainstem masks [16] improve accuracy of group-level analysis and reduce influence of physiological noises on brainstem fMRI. In this study, we examined the functions of human brainstem structures, especially CN and PPN, during walking in a series of bipedal motor tasks in 20 healthy subjects with fMRI. We expected that brainstem activations evoked by simulated gait tasks in humans could be detected by fMRI and functional connectivity and network analysis could reveal the role of brainstem circuits in human walking.

## 2. Materials and Methods

### 2.1. Experimental Design

This is a cross-sectional fMRI study to examine the functions of human brainstem structures during bipedal motor tasks in 20 healthy subjects. Prior to the experiment, a screening form listing conditions that can endanger the safety of subjects during MRI scanning was signed by each subject. The performance of motor tasks was displayed on a video monitor, and a session would be restarted if movements failed to comply with audio cues.

### 2.2. Subjects

We recruited 20 right-handed healthy volunteers (10 males, 10 females) aged 24.5 ± 2.16 years. The experiment was conducted with the approval from the ethic committee of National Research Center for Rehabilitation Technical Aids with No. 20181008. Informed written consent was acquired from each subject. All procedures were performed in accordance with the principles of the Declaration of Helsinki.

### 2.3. Walking Speed Test

The usual walk speed (free gait) of each subject was measured. Subjects were instructed to “walk the way you usually do, as if you were taking a brisk stroll in the park.” Afterward, they walked along a 12 m walkway at a comfortable speed [17]. Each subject repeated this procedure for three times. The first seven strides of the left and right sides in the recorded video (29.97 frames per second) were used to calculate the frequency of free gait for each subject. Here, the start point of the first stride was the heel contact of the first step from standing position because it was impractical to determine the starting time point of the first forward leg movement during gait initiation. We did not measure walking speed after an acceleration period (e.g., 2 m) because in the MRI room during the free-speed condition, a subject was required to start bipedal ankle movement from static condition without an acceleration period.

### 2.4. Motor Task

The fMRI data were collected during six consecutive sessions (one for resting state and five for motor tasks) for each subject. During each session, the subjects were asked to close their eyes, remain alert and awake, and lie on their back with a Siemens pad under their knees to reach a knee-flexed and thigh-supported position for ensuring that the spine was relaxed such that a minimum amount of movement was transferred to the head during the task [18].

The duration of the resting-state session was 480 s (240 volumes) and was always the first session for each subject. The duration of each task session was 390 s (195 volumes). During the second session (the first session among the five types of motor tasks), the subject was asked to alternately move the left or right feet as if performing a self-paced, free-speed walking. The other four tasks were 0.5 Hz, 1 Hz, 2 Hz, and a changing-speed ankle movement; the order of these tasks was counterbalanced for every four subjects in the order of arrival to the MRI room. The subjects were required to perform repetitive alternating ankle dorsiflexion-plantarflexion as if walking. There was no requirement for the range of motion, but the subjects were instructed to follow the audio cue as exactly as they could.

Every task session involved six rest-task cycles comprising 30 s of rest, followed by 30 s of movement with a 30 s rest period at the end. For each task condition, a verbal command “Being ready to lift up your left foot” was delivered with durations of 1.35 and 4 s prior to each task period. For the free-speed condition, movements were self-paced following the audio cue “alternating left and right sides, in a free speed, and keeping a constant speed” that was repeatedly broadcasted (five times during a 30 s period) during each task block. For other task conditions, the movements were paced following the audio cue “left–right–left–right…” with “left” being the first cue. Before MRI scanning of functional images, the subject was trained to dorsiflex the left ankle as the start of bipedal walking-like movement when hearing the first cue “left” and to dorsiflex the right ankle and plantarflex the left ankle when hearing the second cue “right.” A verbal command “stop” was given as the last audio cue to replace “right” during each task block. For the 0.5 Hz condition, there were 15 audio cues during a 30 s period, with eight “left” cues, seven “right” cues, and one “stop” cue in a task block. There were 30 and 60 audio cues in a task block for the 1 and 2 Hz conditions, respectively. For the changing-speed condition, each 30 s task block consisted of 4 audio cues with 1 Hz, 6 cues with 1.5 Hz, 8 cues with 2 Hz, 6 cues with 1.5 Hz, 8 cues with 2 Hz, 8 cues with 1.5 Hz, and 4 cues with 1 Hz in sequence. The average frequency was 1.47 Hz.

Audio commands were broadcasted using the E-Prime software via the intercom system of the MR scanner. The ankle movements of each subject during the free-speed condition were video-recorded (25 frames per second) to calculate the frequency of ankle movement.

During fMRI scanning, an in-house developed system was applied to monitor real-time head movement of the subject. A session would be stopped and restarted if the amplitude of head movements in the *x*-, *y*-, or *z*-axis exceeded 1 mm. Ankle movements were displayed on a video monitor, and a session would also be restarted if ankle movements failed to comply with audio cues.

### 2.5. MRI Data Acquisition

Scanning was performed on a Siemens MAGNETOM Prisma 3T MRI system with a 20-channel coil. Gradient echo images with blood-oxygen-level-dependent (BOLD) contrast were collected using a multi-band sequence (multiband acceleration factor =8, TR 2000 ms, TE 30 ms, flip angle 90°, field of view = 224 mm × 224 mm, matrix size = 112 × 112, voxel size 2.0 × 2.0 × 2.0 mm) consisting of 64 interleaved axial slices with 0.2 mm gaps between slices. T1-weighted images (3D MPRAGE sequence, TR 2530 ms, TE 2.98 ms, flip angle 7°, inversion time 1100 ms, field of view = 256 mm × 256 mm, matrix size = 512 × 512, voxel size 0.5 × 0.5 × 1.0 mm) and field mapping images (TR 635 ms, TE 4.92 and 7.38 ms, flip angle 60°, field of view = 224 mm × 224 mm, matrix size = 112 × 112, voxel size 2.0 × 2.0 × 2.0 mm) were also acquired.

### 2.6. fMRI Activation Analysis

Statistical parametric mapping software (SPM12, Wellcome Trust Centre for Neuroimaging) [19] was used for imaging data processing and activation analysis. BOLD images were corrected for differences in slice acquisition times. A voxel displacement image was generated with the FieldMap Toolbox in SPM and then used for performing realignment and unwarp to correct head motion and geometric distortion. Then, the BOLD images were coregistered to the high-resolution T1 anatomical image. BOLD and T1 images were spatially normalized to standard MNI space as introduced in [15]. This process was guided with a two-stage reference mask to improve brainstem coregistration and normalization. The first stage was a global coregistration and normalization process to the MNI template using affine transformation. The second stage applied a brainstem mask (Supplementary Materials Figure S1) modified from the original mask used in [15] to achieve a brainstem-weighted affine transformation.

The normalized BOLD images were then spatially smoothed using an isotropic Gaussian kernel with a full width at half maximum parameter of 5 mm. Data of the changing-speed condition were smoothed with 3, 4, and 5 mm kernels to demonstrate which one could lead to more sensitive results.

Activation analysis was restricted to the volume inside an anatomical brainstem mask to exclude the most problematic areas adjacent to the brainstem. The boundary voxels between the brainstem and the surrounding tissues such as cerebral spinal fluid (CSF) are susceptible to noises [16]. The brainstem mask was therefore defined on the basis of the averaged T1 image after normalization, and voxels on the boundary of the brainstem were manually removed from the mask as much as possible.

Statistical thresholds were corrected for familywise error (FWE). With a primary threshold of *p* < 0.005, the *p* < 0.05 FWE-corrected cluster extent provided by the SPM toolbox was determined for the whole search volume [20].

The locations of brain activation were defined using the SPM Anatomy Toolbox [21]. The scopes of the CN and the PPN were defined on the basis of the anatomical information [22]. The red nucleus was localized on the basis of averaged functional images of the rest condition from all subjects (Supplementary Materials Figure S2).

### 2.7. Network Analysis

We constructed brain networks for each condition using functional connectivity. The CN, PPN, red nucleus, and pons-medulla junction areas were activated during ankle movements and therefore were selected as nodes in the network. The scopes of the CN, PPN, and red nucleus were defined on the basis of their anatomical information (Supplementary Materials Figure S2). The pons-medulla junction areas commonly activated under all motor conditions (at the *p* < 0.05 threshold with a minimum cluster size of 10 voxels) were divided into four parts, that is, midline areas in the pons or medulla and right or left side of the midline. The midline areas corresponded to the raphe nucleus in the pons or medulla (Supplementary Materials Figure S2).

Cerebral and cerebellar activations commonly detected under all motor conditions (at the same threshold applied for brainstem analysis) were also selected as nodes, with the averaged peak coordinates as node centers and a 3 mm radius sphere shape. The effect size of the averaged BOLD signal changes of voxels within a node was measured using the MarsBaR toolbox (http://marsbar.sourceforge.net), where it is 95% sure that the effect size is greater than 1% of the global mean.

Pearson correlation coefficients between time courses of nodes (average timeseries from non-smoothed data for each condition) were measured using the CONN toolbox (https://web.conn-toolbox.org). To reduce the effects of low-frequency drift and high-frequency noise, the waveform of each brain voxel was filtered using a bandpass filter (0.008 < *f* < 0.09). Realignment parameters were used as first-level covariates. Linear regression was applied to remove signals from ventricular regions, white matter, and their temporal derivatives. Functional connectivity between nodes was represented as edges/links in the weighted undirected graph, and the weight of an edge was the Fisher-transformed correlation coefficient (beta value). The threshold of magnitude of correlations was the false discovery rate-corrected *p* < 0.05.

Network features were analyzed using graph theoretical approaches with the Brain Connectivity Toolbox [23]. The degree of node *i* is the number of edges connected to this node in the network. In a weighted network, each edge carries a numerical value that represents the weight of the edge. The strength of a node is the sum of the weights of all edges linked to this node, which serves as the natural generalization of the degree of node *i* and measures the extent of information transmission between a node and other nodes in the network.

Modules are defined as groups of densely linked nodes that are only sparsely connected to the rest of the network. Hence, nodes within a module achieve a relatively fast rate of information transmission, and different modules perform different functions with some degree of independence [24]. To detect the modularity of each network, the algorithm (modularity und.m in the Brain connectivity toolbox) was repeated for 100 times. The acquired modules were compared with the degree-, weight-, and strength-preserving null models with the same number of nodes [25]. Therefore, for every network, 100 null models were generated with the Brain Connectivity Toolbox. Afterward, 100 results of modularity were calculated for each constructed null model. The modular structure with the highest correlation coefficient value relative to the original solution was identified as the representative of the 100 results because such solution was the closest to the original solution. Finally, 100 representatives from the 100 constructed null models were obtained. The low correlation coefficient values will indicate that the modular structure acquired from the original network is not associated with the weight, degree, and strength properties of the original network.

## 3. Results

### 3.1. Changing-Speed Bipedal Movements Evoked Brainstem Activations

**Figure 1:**
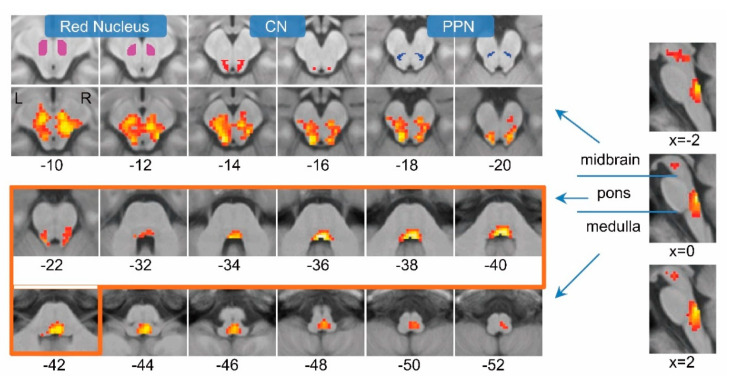
Brainstem activation during the changing-speed locomotion task. Brainstem activation is projected onto the mean T1 image. The numbers in the *x*-axis (for the sagittal plane) or the *z*-axis (for the axial plane) indicate the MNI coordinate. The images of the pons are framed in orange. Brainstem activations in all motor tasks are shown in Supplementary Materials Figure S3. The anatomical locations of the CN, PPN, and red nucleus are shown in the first row (also Supplementary Materials Figure S2).

The 20 subjects were instructed to perform alternating bipedal ankle dorsi-/plantarflexion with speeds of 1, 1.5, and 2 Hz (1.47 Hz on average). We expected such complex simulated-gait movements could evoke relatively high-level brainstem activities. These simulated-gait movements evoked brainstem activations in the CN, PPN, red nucleus, and several pons and medulla areas (Figure 1). This finding determined the role of MLR in human locomotion.

### 3.2. Slow, Fast and Free-Speed Movements also Evoked Brainstem Activations

We further examined bipedal motor tasks with constant audio-paced speeds of 0.5, 1, or 2 Hz to simulate slow and fast locomotion (two well-studied behaviors in animal experiments). A free-speed task (1.46 ± 0.67 Hz) was also used to resemble walking without stress, as a comparison to the more challenging changing-speed task. Brainstem activations were detected under these conditions but often less extensive than those of the changing-speed task (Supplementary Materials Figure S3). Cerebral and cerebellar activations under all motor conditions (at the same threshold applied for brainstem analysis) were presented in Supplementary Materials Figure S4 and Table 1.

Brainstem activations were found in the CN, PPN, pons-medulla junction area, and red nucleus. All these regions play roles in locomotion. CN and PPN neurons send signals to the spinal cord by recruiting neurons in the medulla oblongata [26] or project directly to the spinal cord to control locomotion [6]. The middle parts of the pons-medulla junction area correspond to raphe nuclei in the pons and medulla oblongata, and the lateral parts mainly cover subgroups of nucleus reticularis. In mice, the lower dorsal pons sends projections to hindlimb motor neurons in the spinal cord [27,28], providing a structure path to regulate walking movements. Coordination may be achieved by the activation of the red nucleus via the rubrocerebellar pathways, for example, the dentatorubrothalamic tract [29] (Supplementary Materials Figure S2).

### 3.3. Brainstem Contained Two Modules

Neural tract-tracing techniques reveal pathways between the MLR and other brain regions in animal experiments but are unfeasible in humans. To examine the interactions among brain regions, we constructed a network for each condition with the same group of regions of interest (ROIs) and functional connectivities between each pair of ROIs/nodes. Besides the brainstem nuclei, cerebral and cerebellar activations commonly detected under all motor conditions (at the same threshold applied to brainstem analysis) were selected as ROIs with averaged peak coordinates (Table 1 and Supplementary Materials Table S1) as centers using a 3 mm radius sphere shape.

**Figure 2:**
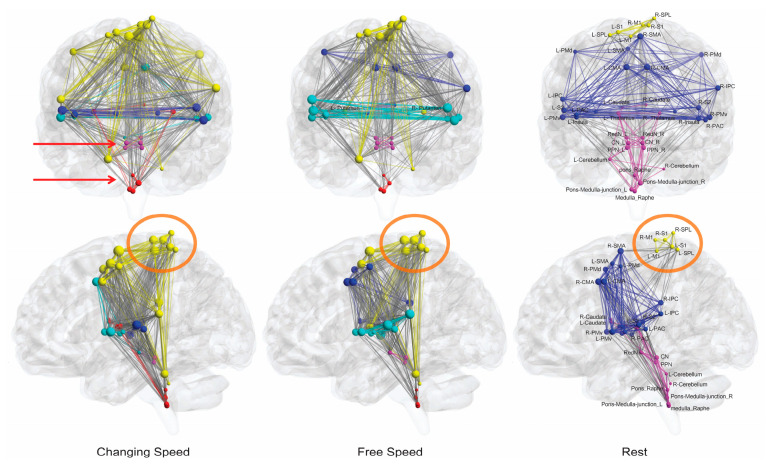
Networks in anatomical space. Edges are sized according to their weight, and brain regions are sized according to nodal strength (the sum of weights of all edges connected to this region). The smaller size of nodes and finer edges in the sparser resting-state network indicate lower nodal strength, weaker internodal connections, and lesser interactions. The brain regions in the same module are indicated in the same color. Bilateral midbrain nodes and pons–medulla nodes constitute two separate modules (red arrows) under all motor task conditions (see Supplementary Materials Figure S5 for all conditions and Supplementary Materials Figure S6 for network layout). The M1–S1–SPL group (orange circles) constitutes a module during resting state that extends to other brain regions in all task conditions. There are no links between this group and brainstem nodes during the resting state (shown in the second row of the rightmost column).

**Figure 3:**
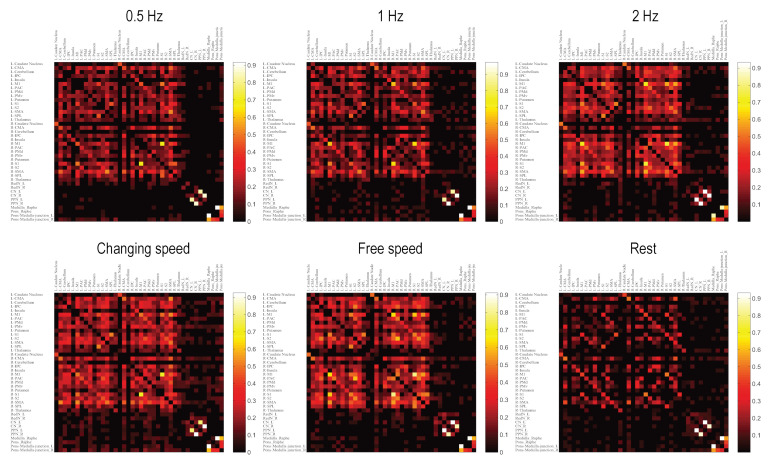
Matrix of edge magnitudes between all pairs of brain regions. The brightness of a grid represents the weight value of the edge between two nodes from the corresponding row and column. The color bar indicates Fisher-transformed correlation coefficient values. Black grids indicate no existing links. The dark area in each network reflects sparser interaction between inside- and outside-brainstem nodes.

The first finding of connectivity/network analysis was brainstem modules. In a network, modules are groups of densely interconnected nodes that are only sparsely connected with the remaining nodes in the network. Bilateral CN–PPN and the four pons–medulla regions constituted two separate modules under all motor conditions, presenting two brainstem functional units for locomotion control (Figure 2, Figures S5 and S6). Outside- and inside-brainstem nodes were connected more densely although the links between the two groups were sparse (Figure 3).

### 3.4. Walking Automaticity Existed during Fast and Free-Speed Walking

The second finding of network analysis supported the existence of walking automaticity during the 2 Hz condition. Behaviorally, pedestrians commonly engage in other activities while walking [30]. Individuals can control a steady-state walking with only minimal use of attention-demanding executive resources of the central nervous system, that is, walking automaticity [31]. This process may involve the use of cortical regions [32] and lumbar spinal circuits producing automatic walking-like muscular activities [33]. In the human brainstem, however, the mechanism of walking automaticity is still unclear.

The primary auditory cortex (PAC) provides timing information to pace bipedal movements. More complex changing-speed tasks present more PAC–CN/PPN edges than other conditions (7 vs. 0–4), indicating the role of PAC–CN/PPN interaction in speed control. Among tasks with constant audio-paced speeds (0.5, 1, and 2 Hz), only the 2 Hz condition had no PAC–CN/PPN edges; thus, the CN and the PPN did not rely on input audio signals to set a gait pace (e.g., a sign of automaticity) because the timing information may be automatically integrated into forward motor planning at a fixed walking speed [34] and fast rhythmic movements are more likely related to automaticity due to weakened cognitive surveillance [35].

Automatic control of the 2 Hz task was also supported by changes in edge numbers between the brainstem and other brain regions. Inside- and outside-brainstem nodes constitute two subnetworks. The edge numbers between the two subnetworks reflect how closely they interact with each other. At constant audio-paced speeds (0.5, 1, and 2 Hz), the edge number of the entire network increased gradually, so did the edge numbers in the inside- and outside-brainstem subnetwork. As the only exception, fewer edges were noted between the two subnetworks in the 2 Hz condition than in the 1 Hz condition (Supplementary Materials Figure S7). The mismatch, a faster speed but a weaker brainstem–cortex interaction, supports a partly “automatic” gait control mechanism by the brainstem. Such automaticity was also seen at the free-speed condition, which was faster than 1 Hz but had a weaker brainstem–cortex interaction (Supplementary Materials Figure S7).

When examining the mechanisms of walking automaticity in the 2 Hz and free-speed tasks, the two conditions were found to commonly present an extended CN–PPN module containing bilateral red nucleus (and even bilateral thalamus in the 2 Hz condition) (Supplementary Materials Figure S5). Thus, this extended CN–PPN–red nucleus module (i.e., stronger interactions among these nodes) appears to be a neural substrate of automatic gait control.

### 3.5. Changing-Speed Condition Had Lower Cortical Load but Higher Brainstem Load Than the Free-Speed Task

As a more complicated task, the changing-speed condition had a similar frequency to the free-speed condition (1.47 Hz vs. 1.46 Hz). Compared with the free-speed task, the changing-speed condition had lower cortical load (averaged effect size of cerebral and cerebellar ROIs 1.62 ± 0.96 vs. 1.84 ± 1.09, *p* = 0.021; paired *t*-test, two tails) but higher brainstem load (averaged effect size of brainstem ROIs 0.23 ± 0.04 vs. 0.14 ± 0.07, *p* = 0.002; paired *t*-test). Such segregation (higher brainstem involvement but reserved cortical resources) can facilitate cognitive function processing in the cortex under complex conditions, thereby providing a neural basis for handling motor-cognitive dual-task walking, such as talking while walking or avoiding obstacles on a pavement in a city center [30].

### 3.6. Interactions between Topmost Brain Regions and Brainstem Existed Only during Motor Conditions

The resting-state network was the most sparse (minimum edge numbers) and had the lowest nodal strength values (Figure 2 and Figure 3), reflecting closer interactions among brain regions at movement states than at resting state. Intriguingly, in the resting-state network, the topmost module composed of bilateral M1, S1, and SPL nodes had no connections to the bottom nodes in the brainstem and cerebellum. All nodes in the topmost module were located at the primary sensorimotor cortex or near regions (Figure 2), indicating that motor execution is the primary role of this module [36]. In all movement conditions, the right M1 was always the region to present the highest response at the beginning of the task condition (Supplementary Materials Figure S8), suggesting its role as the primary mover among all cortical regions in initiating the bipedal movement. Additionally, most other nodes from the same module (right S1, right SPL, and left M1) were in the front ranks among all nodes (Supplementary Materials Figure S8). Therefore, this module mainly initiates the bipedal movements. In task conditions, this module extends to other brain regions, especially the distant cerebellum (Figure 2 and Figure S5), which is in line with the role of the cerebellum in coordinating bipedal movements. Notably, under all movement conditions, links were noted between the M1–S1–SPL group and brainstem circuits, whereas no such connections were observed under the resting state (Figure 2 and Figure 3). As a common feature during bipedal walking, such interactions may rely on ascending and descending projections between the brainstem and the cerebral hemispheres and reserved cerebral planning and cognitive resources while walking, thereby facilitating automatic gait control and dual-task walking.

### 3.7. CN Initiated High-Speed Locomotion but PPN Appeared to Be a “Metronome”

When examining the edges between the right M1 and the CN/PPN at constant audio-paced speeds (0.5, 1, and 2 Hz), the 2 Hz condition only evoked the R_M1–CN connection, and the slower 1 Hz condition only evoked the R_M1–PPN connection (Figure 3), providing a path where CN initiates high-speed locomotion and is thus responsible for rapid escape behavior.

The different roles of the CN and the PPN in locomotion control were also examined by analyzing the levels of signal changes. The behavior of the CN, medulla raphe nucleus, and lateral junction areas was analog to an “all or nothing” operation (Supplementary Materials Figure S9A). That is, these regions maintained a very low effect size at slow speeds (0.5 Hz vs. 1 Hz: *p* = 0.9197; paired *t*-test, two tails) but “jumped” to a higher level during rapid movement (0.5 Hz vs. 2 Hz: *p* = 0.0232; 1 Hz vs. 2 Hz: *p* = 0.0261; paired *t*-test, two tails). The CN group appeared to be nearly “silent” unless a high speed is needed. This result is in line with the notion that the CN is able to elicit high-speed locomotor activity and is thus responsible for rapid escape behavior in non-human vertebrates [5,6]. The effect size of the PPN (and pons raphe nucleus) gradually elevated when the movement speed increased (linear correlation with R^2^ ranging from 0.94 to 1, Supplementary Materials Figure S9B). Therefore, the PPN appears to be a “metronome” inside the brainstem to set a speed for paced bipedal walking.

## 4. Discussion

Through analyzing different simulated-gait tasks, our findings support that the CN is responsible for high-speed locomotion, whereas the PPN appears to be a “metronome” that sets the gait speed during bipedal walking. The brainstem regions participating in controlling bipedal locomotion in humans constitute a module as the neural substrate for walking automaticity. Additionally, during complex walking conditions, we found higher levels of brainstem involvement but reserved cortical resources that provide a neural basis for dual-task walking.

The risk of dementia [1] and falls in the elderly [2] is related to gait disturbance. Brainstem networks are pivotal in motor/sensory functions including the gait control [37,38]. Clarification on the role of brainstem regions in locomotion control can facilitate developing therapeutic interventions targeting CN and PPN or related neural circuits to improve gait control in the elderly with risks of dementia and falls or in patients with a damaged gait such as those suffering from Parkinson’s disease [3] and stroke [4].

We used a masked analysis in this study. BOLD signals of the brainstem and spinal cord are easily contaminated by multiple sources of physiological noises from the pulsatile motion of arteries, the flow of CSF driven by cardiac pulsation and respiratory circle, B- or C-waves, and fluctuations in blood CO2 concentration [39,40]. The arteries and CSF around the brainstem are the main sources of physiological noises in brainstem fMRI. Applying the brainstem mask to exclude areas with high physiological noises is an effective approach to detect signal changes in the brainstem and avoid interference from neighboring physiological noises [16]. However, the sphenoid sinus and the body of the sphenoid are located anterior to the brainstem, thereby resulting in susceptibility-induced BOLD sensitivity losses of the medulla and especially ventral pons (presenting a dark zone in this area) in functional images [41]. Thus, fMRI cannot reliably measure the brainstem function in these regions. We therefore did not include ROIs in the ventral pons and medulla during connectivity analysis. Additionally, because the brainstem mask did not contain voxels in the interface between the brainstem and nearby tissues, real activations of these voxels may be “erased.” For example, the voxels in the region of locus coeruleus lying in the margin of the brainstem were not included in the mask for activation analysis. Similarly, periaqueductal grey was heavily influenced by the pulsation of CSF inside the midbrain aqueduct and therefore was not selected as an ROI.

Smoothing with a 5 mm Gaussian kernel resulted in larger midbrain activations than those with smaller kernels (Supplementary Materials Figure S3B). The sensitivity of combining the same normalization method [15] as applied in this study and a similar smooth kernel (4.5 mm) was also proven by [42].

This study has several possible limitations. Firstly, the subjects performing the simulated-gait tasks experienced a different body position, visual field, and somatosensory feedback compared with real walking. Thus, one may doubt that the evoked brainstem function was probably different from that during real locomotion to some extent. However, on the basis of previous fMRI studies [18,43,44] adopting ankle movement as a critical component of gait cycle, we applied simulated-gait tasks with bipedal movement. The fundamental components of human locomotion including the locomotor rhythm, alternating intralimb coordination between flexor and extensor, and coordination between the left and right lower limbs [33] are involved in the simulated-gait tasks. Secondly, a previous study indicates that the medio-caudal part of the MLR in humans involves in gait control whereas the latero-rostral part involves in balance [45]. Although the scopes of the CN and the PPN could be separated in this study, a limited spatial resolution provided by fMRI was insufficient to differentiate subgroups of neurons within the PPN or the CN detected in mice [6] or humans [22]. Thirdly, the spreading distance of neuronal activity-induced responses of microcirculation is approximately 3–5 mm [46]. The adjacency of brainstem nodes in the pons and medulla (or in the midbrain) may therefore interfere with functional connectivity measurement among nearby nodes, for example, the risk that two adjacent nodes not only present high degree of correlation but share identical links to other regions. However, our results showed that the nodes close to each other exhibited different connecting patterns. For example, the left CN and the left PPN are adjacent to each other, but only the former had connection with the right SMA (Figure 3). Such difference possibly stems from measuring functional connectivity by using unsmoothed data with the CONN toolbox and the voxels in a node departing from the internodal border.

## 5. Conclusions

We detected brainstem activations evoked by simulated gait tasks in humans by using fMRI. Activations covered CN, PPN, and other brainstem regions. These regions appear to be the neural substrate for walking control in the human brainstem.

## Figures and Tables

**Table 1:** Peak brain activations.

Brain Areas	0.5 Hz				1 Hz				2 Hz				Changing Speed				Free Speed			
	MNI Coordinates			*T* Value	MNI Coordinates			*T* Value	MNI Coordinates			*T* Value	MNI Coordinates			*T* Value	MNI Coordinates			*T* Value
L_SMA	−4	8	45	9.25	−8	6	53	12.73	−6	4	56	11.05	−4	6	49	11.06	−2	−18	73	11.65
R_SMA	4	−10	64	10.81	2	−10	67	11.74	2	−10	64	11.00	6	8	64	9.70	4	−4	62	11.35
L_M1	−2	−28	64	5.42	−2	−28	62	7.16	−2	−28	64	10.28	−2	−30	62	9.22	−6	−42	69	12.16
R_M1	6	−26	71	5.79	6	−26	69	5.51	6	−38	75	8.35	6	−22	69	7.65	8	−36	75	11.16
L_CMA	−4	4	45	7.65	−4	4	45	7.15	−6	10	42	6.91	−8	10	42	7.88	−10	8	36	9.03
R_CMA	12	4	40	7.51	12	8	45	6.91	14	16	36	7.04	4	14	45	8.32	4	14	45	7.85
L_PMd	−14	−18	73	6.52	−52	−2	51	5.11	−50	−2	45	7.85	−50	−4	47	8.94	−48	0	51	6.75
R_PMd	50	4	51	6.47	50	2	51	8.53	48	6	53	7.80	40	6	51	8.21	52	10	49	8.90
L_PMv	−50	0	7	8.77	−54	2	5	6.90	−48	0	7	9.69	−54	2	5	8.10	−52	2	7	9.06
R_PMv	52	8	12	8.56	48	6	9	5.49	56	4	3	9.08	56	4	3	8.52	58	4	5	7.65
L_S1	−12	−44	73	4.39	−12	−42	67	3.33	−12	−44	67	6.78	−12	−44	67	6.64	−12	−36	62	9.49
R_S1	12	−36	71	4.49	12	−36	71	3.81	8	−40	75	7.31	10	−36	71	6.59	10	−36	71	9.24
L_SPL	−18	−48	67	4.74	−12	−48	80	3.46	−12	−42	62	6.12	−34	−54	47	6.71	−16	−38	69	8.49
R_SPL	16	−46	78	6.26	16	−46	80	4.81	10	−40	75	6.56	12	−40	75	6.55	14	−42	75	8.90
L_IPC	−60	−24	23	6.10	−50	−34	16	7.75	−50	−36	20	8.70	−50	−38	20	10.13	−52	−36	18	11.85
R_ IPC	60	−34	25	6.24	58	−30	23	6.52	50	−28	29	10.96	62	−46	36	10.24	68	−30	20	8.59
L_S2	−50	0	7	8.77	−60	−28	14	8.41	−50	−12	7	11.57	−56	−28	14	10.83	−38	−24	14	12.27
R_S2	32	−24	16	6.32	44	−22	12	7.14	46	−22	12	11.55	66	−18	14	14.62	44	−20	12	15.96
L_PAC	−40	−22	3	5.77	−40	−28	12	4.96	−40	−28	12	10.23	−48	−10	3	10.59	−40	−28	12	15.45
R_ PAC	52	−12	5	3.87	50	−10	3	5.49	56	−10	−2	8.64	50	−12	5	10.55	48	−20	9	15.23
L_mid-posterior insula	−42	2	5	9.45	−44	0	5	5.56	−46	−10	3	7.22	−46	−10	3	7.22	−34	−24	16	12.01
R_mid-posterior insula	46	4	3	7.99	48	−6	1	5.31	50	−4	3	7.79	50	−4	3	8.12	38	−20	14	8.39
L_anterior insula	−36	18	7	5.22	−34	−8	5	5.32	−38	16	7	9.44	−36	18	7	10.02	−28	20	9	6.09
R_anterior insula	42	22	3	3.74	34	22	7	3.5 *	34	18	9	7.62	32	26	3	7.75	34	26	5	5.66
L_Caudate Nucleus	−14	4	12	6.14	10	2	12	6.09	−18	0	20	5.79	−14	0	14	7.59	−10	4	7	7.01
R_Caudate Nucleus	10	8	14	6.92	−16	4	14	7.06	16	−4	18	6.24	18	0	18	7.08	14	4	12	6.21
L_Putamen	−28	−4	12	11.33	−26	0	12	9.04	−30	−8	7	8.19	−26	−2	14	8.79	−30	−14	7	10.95
R_Putamen	28	−4	14	10.00	28	0	9	9.63	30	−4	7	9.6	30	−6	9	10.03	30	−4	12	10.05
L_Thalamus	−8	−20	3	9.72	−6	−18	3	8.28	−14	−16	14	8.48	−14	−14	9	10.85	−16	−18	14	11.22
R_Thalamus	14	−8	7	9.45	16	−14	9	9.39	20	−16	14	10.58	18	−18	14	11.14	22	−22	3	8.09
L_Cerebellum	−22	−38	−26	10.21	−22	−38	−26	13.59	−16	−40	−24	12.72	−16	−38	−21	15.69	−16	−38	−21	13.13
R_Cerebellum	26	−38	−30	8.41	20	−38	−24	13.23	18	−38	−24	11.56	22	−38	−24	14.68	18	−52	−54	6.77

* Under the threshold *p* < 0.005. For others, the threshold is at *p* FWE < 0.05; see the Materials and Methods section for details. Peak activations with a zero value in the *x*-axis were not included. The posterior insula was shown because each condition did not consistently evoke activations in the anterior insula. R: right, L: left; CMA: cingulate motor area; IPC: inferior parietal cortex; M1: primary motor cortex; PAC: primary auditory cortex; PMd: dorsal premotor cortex; PMv: ventral premotor cortex; S1: primary somatosensory area; S2: secondary somatosensory area; SMA: supplementary motor area; SPL: superior parietal lobule.

## Data Availability

Activation maps and connectivity matrices can be found in Figshare: 12732125.

## Supplementary Materials

The following are available online at https://www.mdpi.com/2076-3425/10/10/757/s1, Figure S1: Masks for coregistration and activation analysis; Figure S2: Location of brainstem nucleus; Figure S3: Brainstem activation evoked by all motor tasks; Figure S4: Cerebral and cerebellar activations; Figure S5: Networks in anatomical space (all conditions); Figure S6: Layout of networks; Figure S7: Mismatch between gait speed and edge numbers; Figure S8: Fitted responses of the task condition; Figure S9: Contrast estimates of CN and PPN; Figure.S10: Flow chart of the fMRI experiment; Table S1: Locations of node centers.
