# Supplementary material for: Functional MRI Reveals Locomotion-Control Neural Circuits in Human Brainstem
Source: Brain Sci. 2020 Oct 20;10(10):757. doi: 10.3390/brainsci10100757 (PMC7589833; doi:10.3390/brainsci10100757)
Supplement: Supplementary file 1 [file brainsci-10-00757-s001.pdf]

Functional MRI reveals locomotion-control neural circuits in human brainstem

Pengxu Wei [1,2], Tong Zou [2], Zeping Lv [1,2] and Yubo Fan [1,2*]

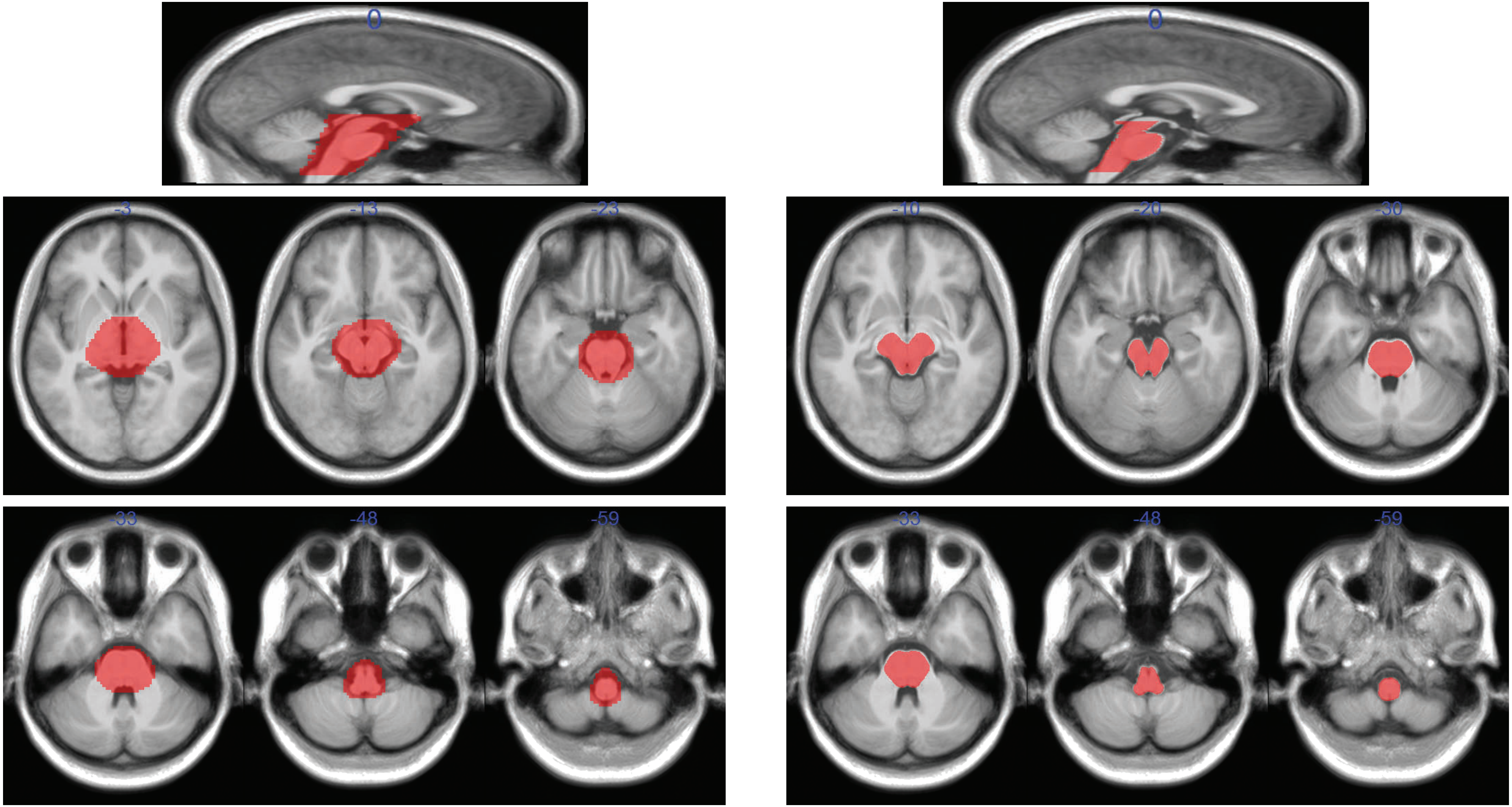

Co-registration mask

Activation-detection mask

Fig. S1. Masks for coregistration and activation analysis.

The coregistration mask was modified from the mask in [1] and incorporated the brainstem and nearby CSF (from z = -3 to z =-62). This mask weighed the cost function in the affine coregistration to improve brainstem coregistration and normalization. With reference of the coregistration mask, the activation mask was created by cutting off outside-brainstem areas and voxels in the border of the brainstem (from z = -10 to z = -59).

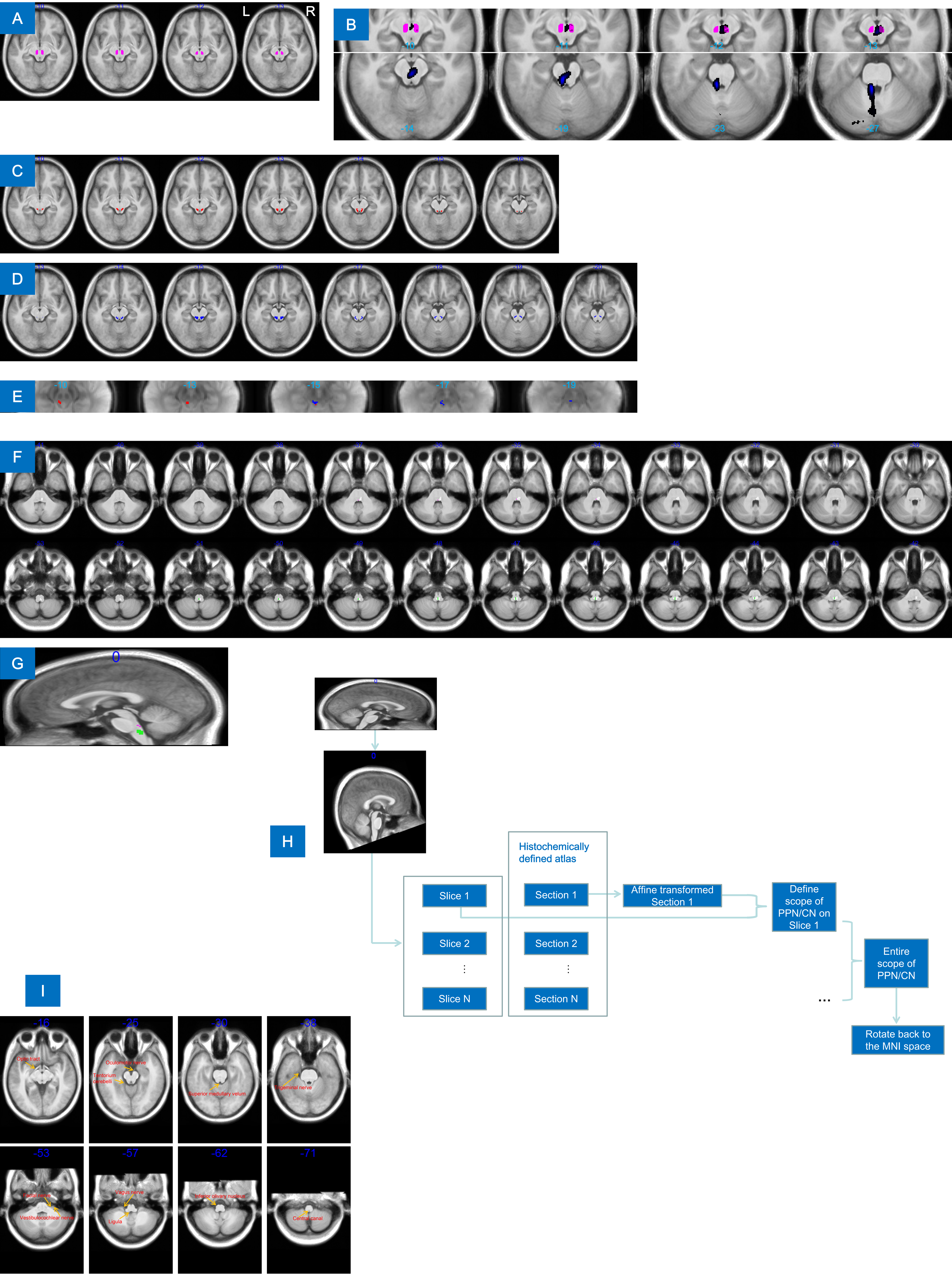
A
L
R
B
C
D
E
F
G
H
Histochemically defined atlas
Slice 1
Slice 2
Slice N
Section 1
Section 2
Section N
Affine transformed Section 1
Define scope of PPN/CN on Slice 1
Entire scope of PPN/CN
Rotate back to the MNI space
I
-16
-25
-30
-38
-53
-57
-62
-71
Optic tract
Oculomotor nerve
Tentorium cerebelli
Superior medullary velum
Trigeminal nerve
Facial nerve
Vestibulocochlear nerve
Vagus nerve
Ligula
Inferior olivary nucleus
Central canal

Fig. S2. Location of brainstem nucleus.

The numbers indicate the MNI coordinates in the x-axis for the sagittal plane or in the z-axis for the axial plane.

(A) The red nucleus is shown in violet and projected onto the mean T1 anatomical image. The scope of the red nucleus was manually defined on the basis of the mean functional images of the rest condition, with examples shown in (E). The iron-rich red nucleus has higher magnetic susceptibility than many gray matter nuclei, such as the dentate nucleus and putamen[2], and present a darker area than surrounding tissues in T2* images as shown in the leftmost slice in (E). A globular shape and a clear boundary of the red nucleus therefore lead to little ambiguity in determining its proper scope[3].

(B) Spatial relation between the red nucleus (in violet) and the cerebello-rubral tract (blue to black) of the superior cerebellar peduncle that runs from a cerebellar hemisphere to the opposite red nucleus, as a validation of the defined scope of the red nucleus. The cerebello-rubral tract is presented using the human probabilistic atlas based on the connectome imaging data[4].

(C) The scope of the CN (in red) was defined on the basis of the anatomical information[5], projected onto the mean T1 anatomical image of all subjects.

(D) The scope of the PPN (in blue) was also defined on the basis of the anatomical information[5], projected onto the mean T1 anatomical image of all subjects.

(E) The scopes of the CN (red) and PPN (blue) were presented onto the mean functional image of the rest condition from all subjects. All slices containing either of the two ROIs are presented. Note that the shape of each ROI changes due to a lower spatial resolution of the mean functional image in comparison with the high-resolution T1 image.

(F) Midline activations in the pons (violet) and medulla oblongata (green) mainly correspond to the raphe nuclei on the basis of [5, 6]. The width was arbitrarily set to 3 mm when defined on the T1 images (i.e., the midline plus both of its sides) and automatically transformed to 2 mm when projected on the functional images due to a lower spatial resolution of these images. Lateral activated regions (white) in the pons–medulla junction zone mainly cover subgroups of the nucleus reticularis on the basis of [5, 6].

(G) The raphe nuclei in the pons and medulla are shown in the sagittal plane in violet and green, respectively.

(H) The flowchart defining the scope of the CN and PPN is shown. The histochemically defined atlas consisted of serial axial sections in an axial plane perpendicular to the long axis of the brainstem. The mean T1 image (in the MNI space) was first rotated to the same spatial orientation as the atlas. Afterward, each section of the atlas containing the PPN and/or CN was matched to a corresponding slice of the T1 images on the basis of anatomical landmarks (I). An affine transform was then applied to the section to match the corresponding slice. The scope of the CN/PPN on the MRI slice was defined on the basis of the transformed section. The scopes from all slices constituted an ROI of CN/PPN, which was rotated to the MNI space.

(I) Example landmarks are presented on the axial plane (after rotation; that is, each axial slice is perpendicular to the long axis of the brainstem). The landmarks were used

to determine which section of the histochemically defined atlas matched to a slice of the T1 image.

Red nucleus activations were found in all rhythmic bipedal movement conditions (0.5 Hz, 1 Hz, 2 Hz, and changing speed,all of which use audio cues to pace movement) but not in the free-speed condition (no rhythmic audio cues to pace movement). Functional connectivities between the red nucleus and the cerebellum were detected in 1 Hz, 2 Hz, and changing-speed tasks but not in the 0.5 Hz and free-speed conditions (Fig. 3). The 0.5 Hz (very slow speed) and free-speed (no rhythmic audio cues) tasks should not be fine-tuned movements requiring strong cerebellum control. We thus propose that such functional connectivities indicate a role of the red nucleus in coordinating ankle movements together with the cerebellum for faster (1 and 2 Hz vs. 0.5 Hz) or more complex (changing-speed vs. free-speed) bipedal movements via the rubrocerebellar pathways (e.g. the dentatorubrothalamic tract) [7].

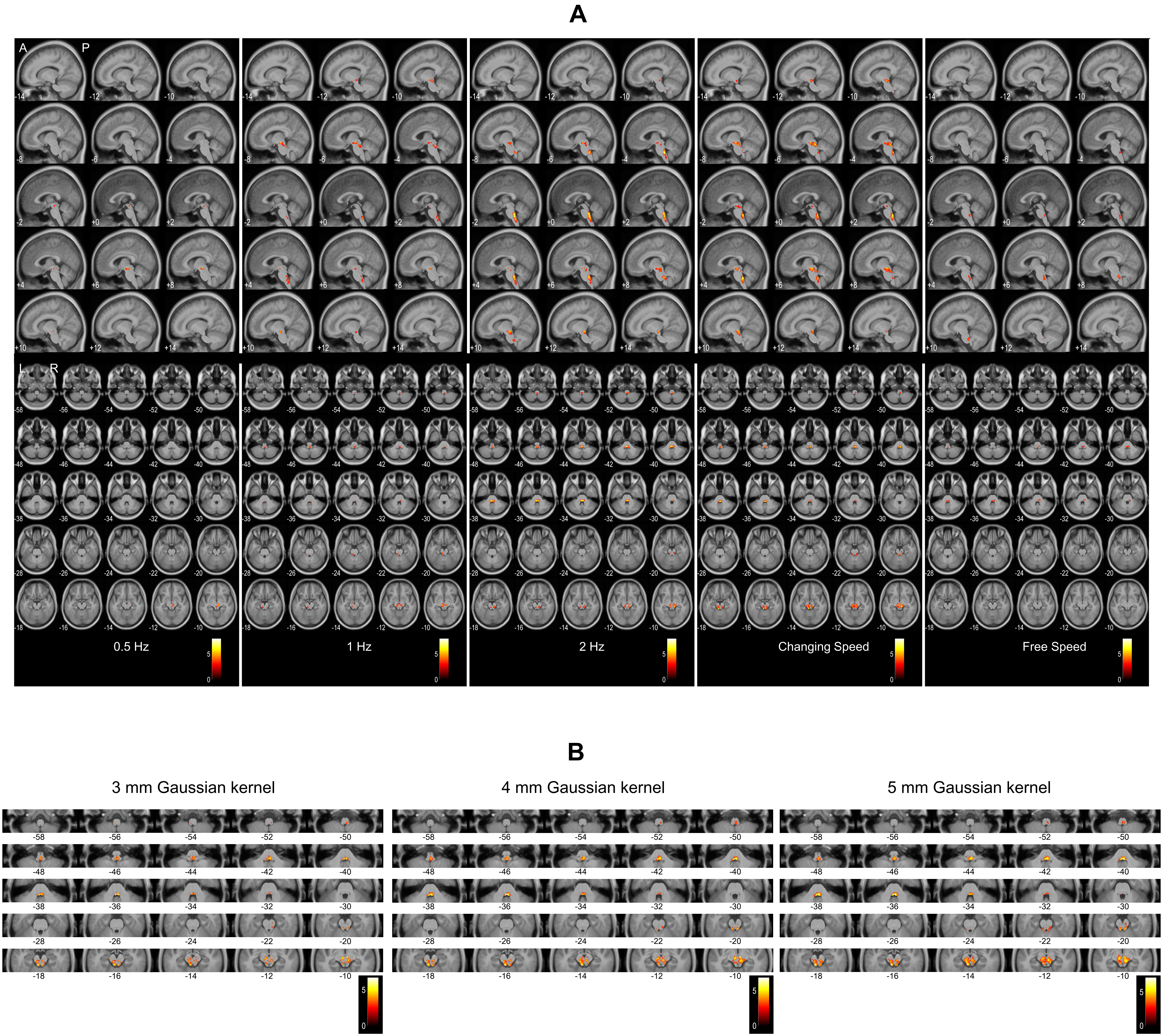
A
A
P
L
R
0.5 Hz
1 Hz
2 Hz
Changing Speed
Free Speed
B
3 mm Gaussian kernel
4 mm Gaussian kernel
5 mm Gaussian kernel

Fig. S3. Brainstem activation evoked by all motor tasks.

(A) Brainstem activation is projected onto the averaged T1 anatomical image of all subjects. The numbers in the x-axis (for the sagittal plane) or the z-axis (for the axial plane) indicate the MNI coordinate (in mm). The locations of the CN, PPN, red nucleus, pons raphe nucleus, medulla raphe nucleus, and right/left sides of the pons–medulla junction are presented in **Fig. S2**.

(B) Brainstem activations with different Gaussian kernels are shown. Data of the changing-speed condition were smoothed with 3, 4, and 5 mm kernels. The activation maps are thresholded under the $p < 0.05$ FWE-corrected cluster extent provided by the SPM toolbox, with a primary threshold of $p < 0.005$. The 5 mm kernel leads to larger activation clusters and therefore appears to be more sensitive. The sensitivity of combining the same normalization method [1] and a similar smooth kernel (4.5 mm) was also proven by [8].

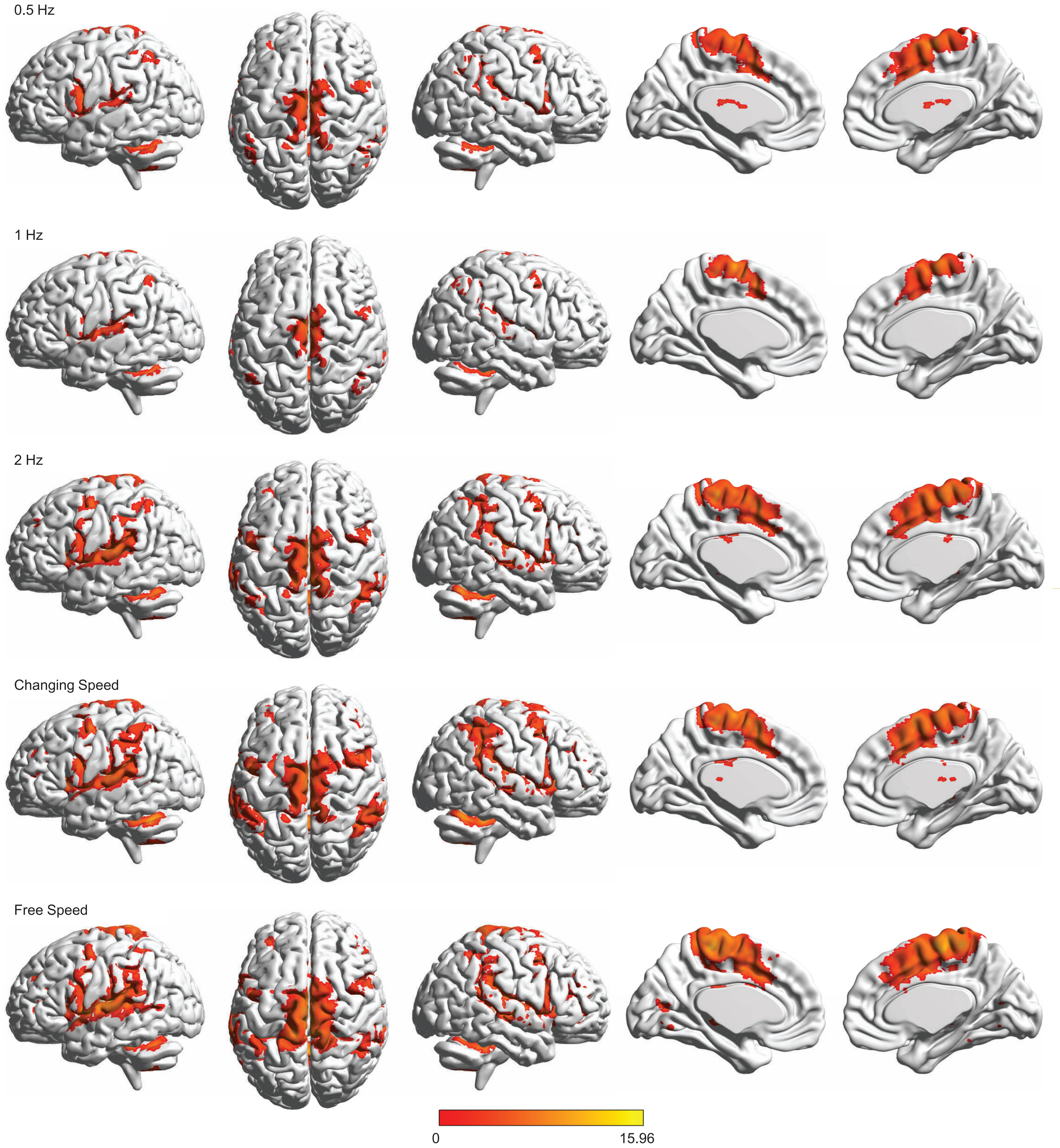
0.5 Hz
1 Hz
2 Hz
Changing Speed
Free Speed
0
15.96

Fig. S4. Cerebral and cerebellar activations.

Cerebral and cerebellar activations evoked by different motor tasks are projected onto the normalized 3D brain with the BrainNet Viewer (http://www.nitrc.org/projects/bnv/). The two rightmost columns are the midsagittal view of the right hemisphere and the left hemisphere. The colored bar indicates *t* values.

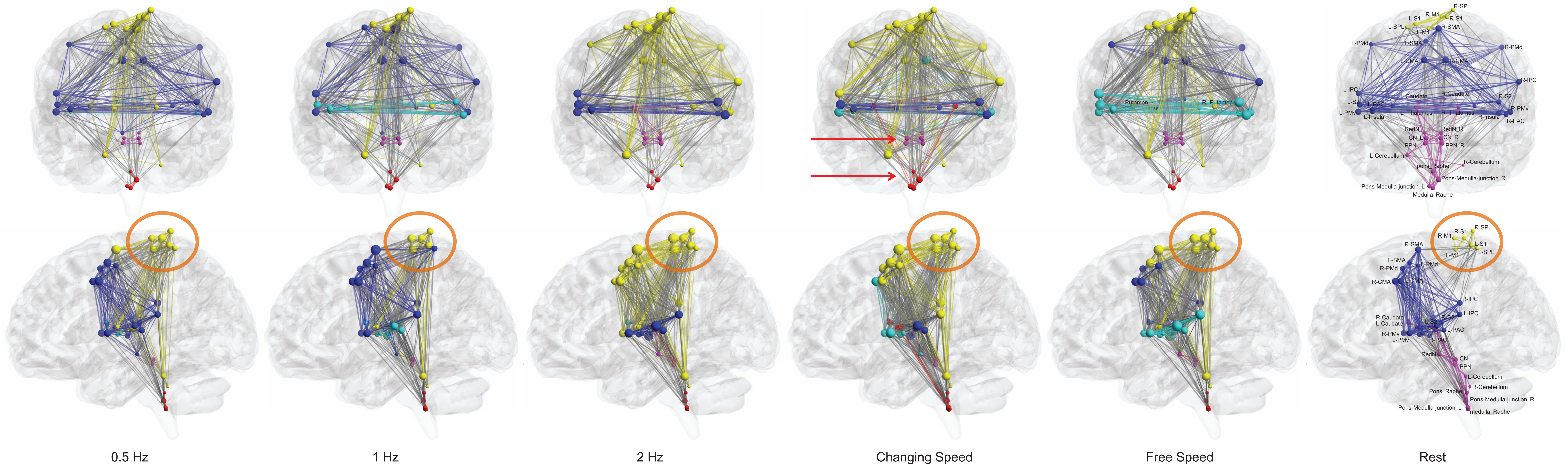
0.5 Hz
1 Hz
2 Hz
Changing Speed
Free Speed
Rest

Fig. S5. Networks in anatomical space (all conditions).

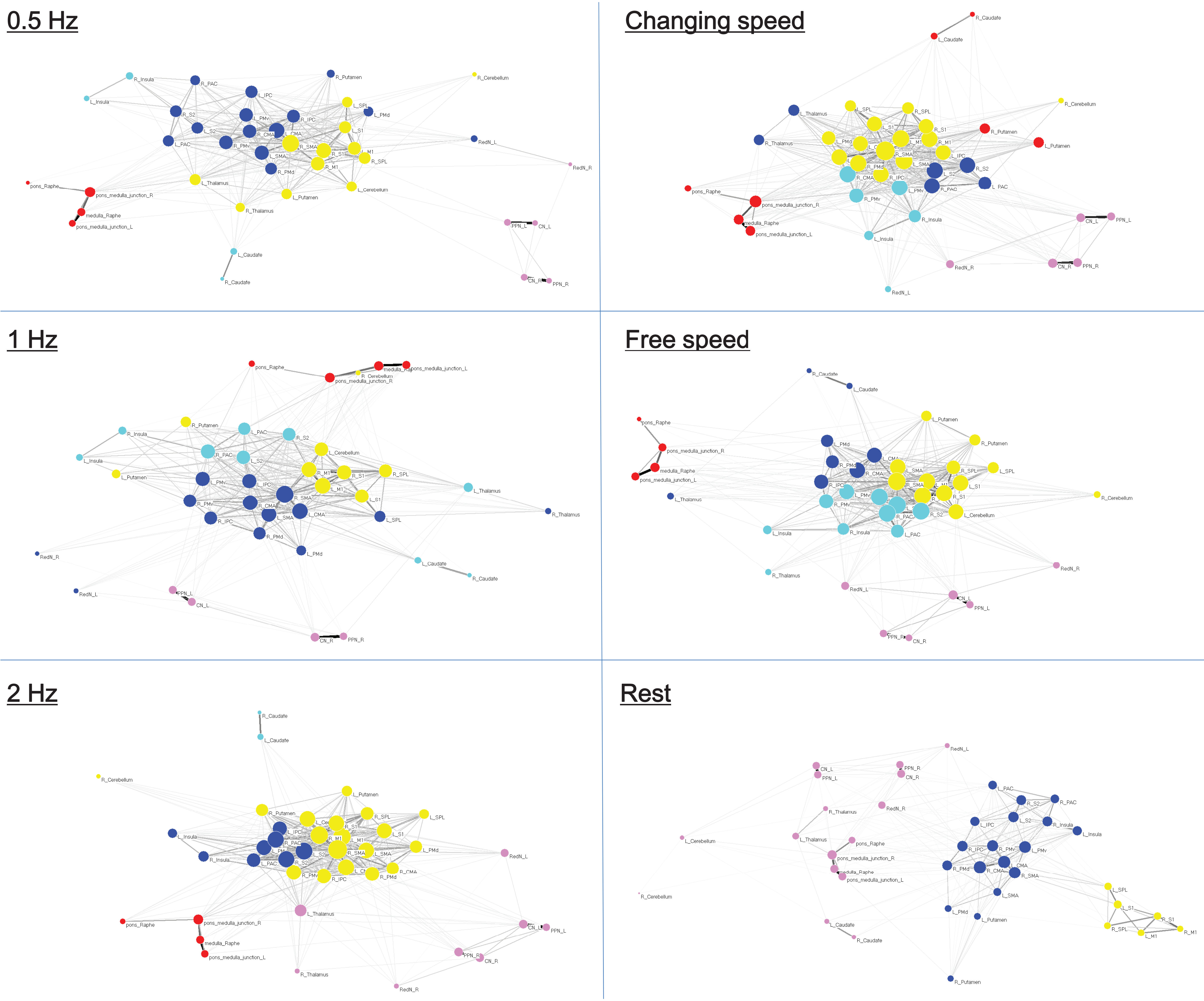
0.5 Hz
R_Insula
L_Insula
R_PAC
L_IPC
R_Putamen
R_Cerebellum
R_S2
L_PMv
R_IPC
L_SPL
L_PMd
L_S2
R_CMA
L_CMA
L_S1
RedN_L
L_PAC
R_PMv
R_SMA
L_SMA
R_S1
L_M1
R_SPL
R_PMd
R_M1
RedN_R
pons_Raphe
pons_medulla_junction_R
L_Thalamus
L_Cerebellum
L_Putamen
medulla_Raphe
R_Thalamus
pons_medulla_junction_L
PPN_L
CN_L
L_Caudate
R_Caudate
CN_R
PPN_R
Changing speed
R_Caudate
L_Caudate
R_Cerebellum
L_Thalamus
L_SPL
R_SPL
L_S1
R_S1
R_Putamen
R_Thalamus
L_PMd
R_M1
L_Putamen
R_SMA
L_IPC
L_SMA
R_PMd
R_S2
R_CMA
R_IPC
L_S2
pons_Raphe
L_PMv
R_PAC
L_PAC
pons_medulla_junction_R
R_PMv
R_Insula
medulla_Raphe
CN_L
PPN_L
pons_medulla_junction_L
L_Insula
RedN_R
CN_R
PPN_R
RedN_L
1 Hz
pons_Raphe
medulla_Raphe
pons_medulla_junction_L
R_Cerebellum
pons_medulla_junction_R
R_Putamen
R_Insula
L_PAC
R_S2
L_Cerebellum
R_PAC
L_Insula
L_S2
R_M1
R_S1
R_SPL
L_Putamen
L_PMv
L_IPC
L_M1
L_Thalamus
R_SMA
L_S1
R_Thalamus
R_PMv
R_CMA
R_IPC
L_SMA
L_CMA
L_SPL
R_PMd
RedN_R
L_PMd
L_Caudate
R_Caudate
RedN_L
PPN_L
CN_L
CN_R
PPN_R
Free speed
R_Caudate
L_Caudate
pons_Raphe
L_Putamen
R_Putamen
pons_medulla_junction_R
L_PMd
L_CMA
R_PMd
L_SMA
R_SPL
L_SPL
medulla_Raphe
R_CMA
pons_medulla_junction_L
R_SMA
R_IPC
L_S1
L_Thalamus
L_PMv
R_S1
R_Cerebellum
R_PMv
R_PAC
R_S2
L_Cerebellum
L_Insula
R_Insula
L_PAC
R_Thalamus
RedN_R
RedN_L
CN_L
PPN_L
PPN_R
CN_R
2 Hz
R_Caudate
L_Caudate
R_Cerebellum
L_Putamen
R_Putamen
R_SPL
L_SPL
L_IPC
R_S1
L_Insula
R_PAC
R_M1
L_M1
L_S1
L_PMd
R_Insula
L_S2
R_SMA
L_SMA
L_PAC
R_S2
R_PMv
R_IPC
R_PMd
R_CMA
RedN_L
L_Thalamus
pons_Raphe
pons_medulla_junction_R
CN_L
PPN_L
medulla_Raphe
pons_medulla_junction_L
PPN_R
CN_R
R_Thalamus
RedN_R
Rest
RedN_L
CN_L
PPN_R
PPN_L
CN_R
L_PAC
R_Thalamus
RedN_R
R_S2
R_PAC
L_IPC
L_S2
R_Insula
L_Insula
L_Cerebellum
L_Thalamus
pons_Raphe
R_IPC
R_PMv
L_PMv
pons_medulla_junction_R
L_CMA
R_PMd
R_CMA
R_SMA
medulla_Raphe
pons_medulla_junction_L
L_SMA
L_SPL
R_Cerebellum
L_S1
L_PMd
L_Caudate
L_Putamen
R_S1
R_SPL
L_M1
R_M1
R_Caudate
R_Putamen

Fig. S6. Layout of networks.

The nodes in the same module are shown in the same color. The nodes/links are sized according to their strength/weight values. Layouts are generated using the Kamada–Kawai force-spring layout algorithm (http://pajek.imfm.si). The visualization method defines the geometric distance between nodes in a network as the summation of edge weights. Every pair of nodes is connected bya "spring" with a desirable length to reach a state, in which the total spring energy of the network is minimal. The final balanced condition is formulated as the square summation of the differences between desirable distances and real ones for all pairs of vertices.

In the resting state, the topmost nodes constitute a module, and more caudally located nodes, including brainstem regions, constitute a module. During task conditions, the former extends to lower brain regions, such as at least one of two cerebellar nodes, whereas the brainstem nodes constitute two separate modules, that is, a CN–PPN (midbrain) group and a pons–medulla group.

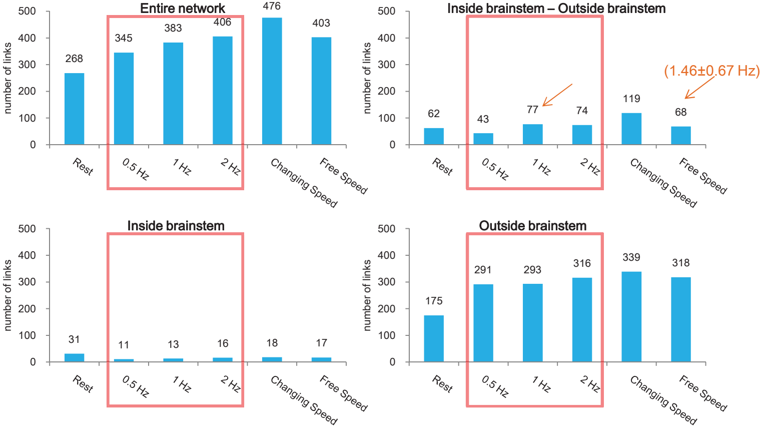
Entire network
number of links
500
400
300
200
100
0
268
345
383
406
476
403
Rest
0.5 Hz
1 Hz
2 Hz
Changing Speed
Free Speed
Inside brainstem – Outside brainstem
62
43
77
74
119
68
(1.46±0.67 Hz)
Inside brainstem
31
11
13
16
18
17
Outside brainstem
175
291
293
316
339
318

Fig. S7. Mismatch between gait speed and edge numbers.

At constant audio-paced speeds (0.5, 1, and 2 Hz, highlighted with frames), the edge number of the entire network, the inside-brainstem subnetwork, and the outside-brainstem subnetwork increased gradually. However, fewer edges were present between the inside- and outside-brainstem subnetworks in the 2 Hz condition than those in the 1 Hz condition, presenting a mismatch between the behavior (faster speed) and the network feature (less interactions between brainstem and cortical structures). Such mismatch is also seen when comparing the 1 Hz and free-speed conditions (highlighted with arrows).

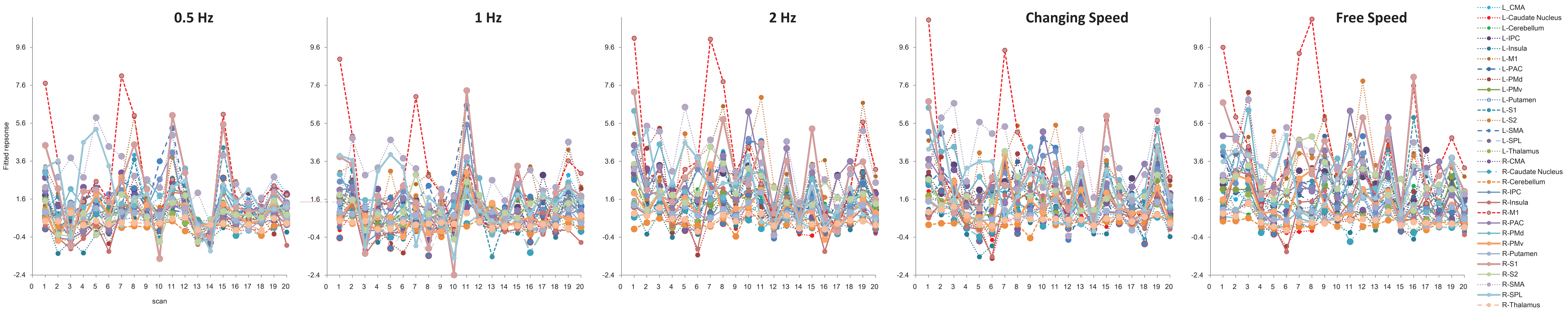
0.5 Hz
1 Hz
2 Hz
Changing Speed
Free Speed
Fitted repsonse
scan
9.6
7.6
5.6
3.6
1.6
-0.4
-2.4
0 1 2 3 4 5 6 7 8 9 10 11 12 13 14 15 16 17 18 19 20
L_CMA
L-Caudate Nucleus
L-Cerebellum
L-IPC
L-Insula
L-M1
L-PAC
L-PMd
L-PMv
L-Putamen
L-S1
L-S2
L-SMA
L-SPL
L-Thalamus
R-CMA
R-Caudate Nucleus
R-Cerebellum
R-IPC
R-Insula
R-M1
R-PAC
R-PMd
R-PMv
R-Putamen
R-S1
R-S2
R-SMA
R-SPL
R-Thalamus

Fig. S8. Fitted responses of the task condition.
Acquired using the MarsBaR toolbox, the fitted responses (move > rest) of each ROI across subjects are displayed. The x-axis indicates scan numbers, and the y-axis indicates the fitted response. Note that the right M1 always presents the highest response at the beginning of the task condition across all types of movements. Cerebral and cerebellar activations in the anatomical space are shown in **Fig. S4**.

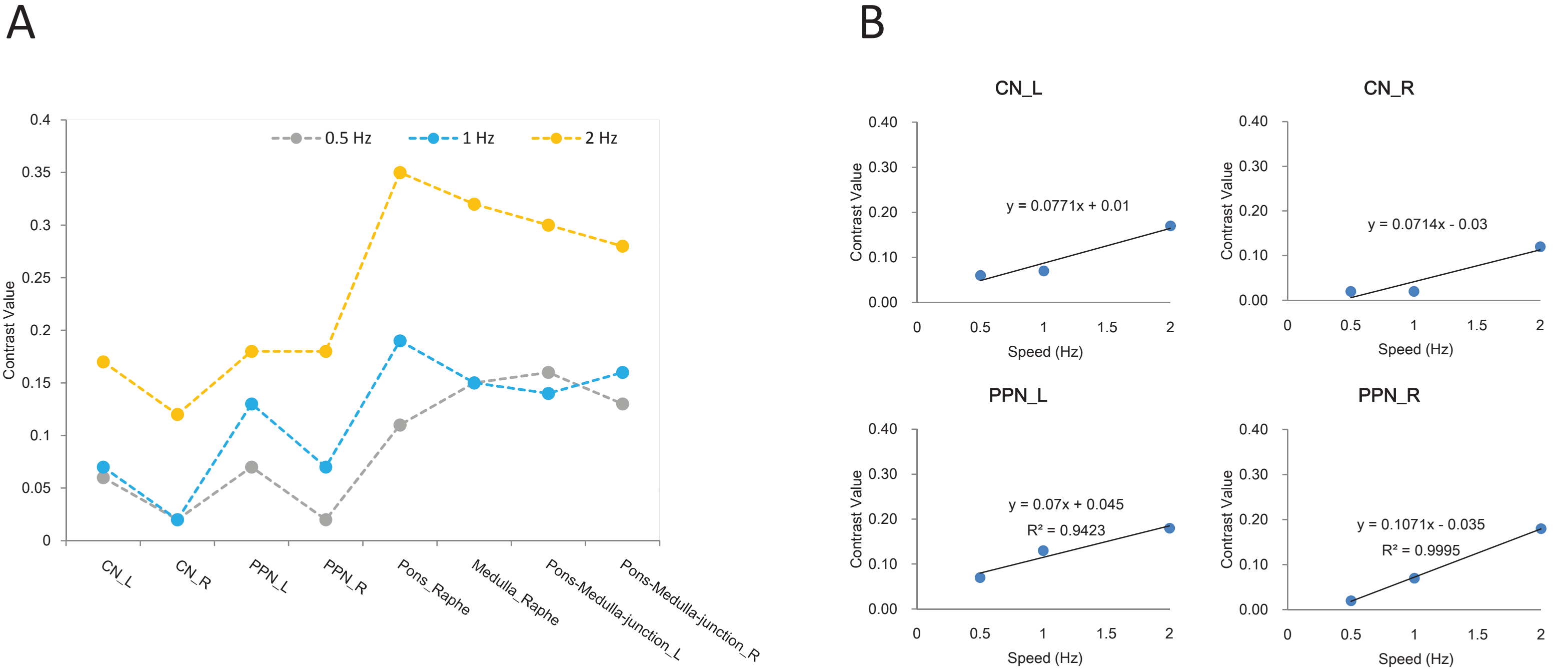
A
0.5 Hz
1 Hz
2 Hz
Contrast Value
0.4
0.35
0.3
0.25
0.2
0.15
0.1
0.05
0
CN_L
CN_R
PPN_L
PPN_R
Pons_Raphe
Medulla_Raphe
Pons-Medulla-junction_L
Pons-Medulla-junction_R
B
CN_L
y = 0.0771x + 0.01
CN_R
y = 0.0714x - 0.03
PPN_L
y = 0.07x + 0.045
R² = 0.9423
PPN_R
y = 0.1071x - 0.035
R² = 0.9995
Contrast Value
0.40
0.30
0.20
0.10
0.00
Speed (Hz)
0
0.5
1
1.5
2

Fig. S9. Contrast estimates of CN and PPN.

(A) Acquired using the MarsBaR toolbox, the effect size of the CN and PPN of the constant audio-paced speeds (0.5, 1, and 2 Hz)is presented. The CN maintains at a very low level at slow movements but jumps to a higher level, being analogue to an “all or nothing” operation. The effect size of the PPN elevated gradually.

(B) Data are plotted with fitting curves of the linear regression equation and coefficient of determination R^2. The changes of bilateral PPN coincide with the fitting curve, but those of the CN do not.

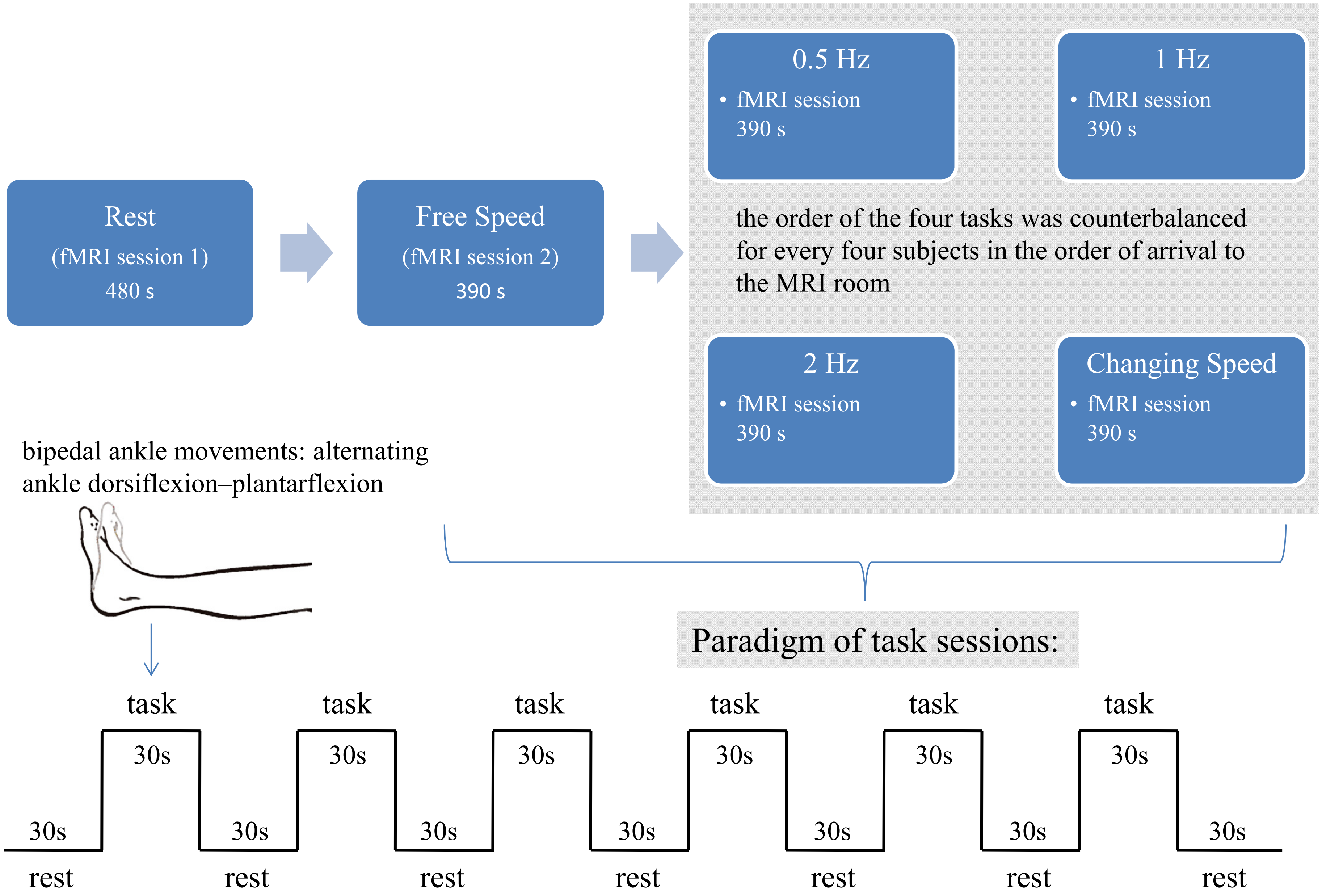
Rest
(fMRI session 1)
480 s
Free Speed
(fMRI session 2)
390 s
0.5 Hz
• fMRI session
390 s
1 Hz
• fMRI session
390 s
the order of the four tasks was counterbalanced for every four subjects in the order of arrival to the MRI room
2 Hz
• fMRI session
390 s
Changing Speed
• fMRI session
390 s
bipedal ankle movements: alternating ankle dorsiflexion–plantarflexion
Paradigm of task sessions:
task
30s
rest
30s

Fig. S10. Flow chart of the fMRI experiment.

Table S1 Locations of node centers.

Centers of nodes	MNI coordinates		
L_Caudate	-9 2 13		
L_CMA	-6 7 42		
L_Cerebellum -	18	-38	-24
L_IPC -	52	-34	19
L_Insula -4	2	-8	6
L_M1 -	3	-31	64
L_PAC -	42	-23	8
L_PMd -	43	-5	53
L_PMv -	52	1	6
L_Putamen -	28	-6	10
L_S1 -	12	-42	67
L_S2 -	51	-18	11
L_SMA	-5 1 55		
L_SPL -	18	-46	65
L_Thalamus -	12	-17	9
R_Caudate 8		2	15
R_CMA 9		11	42
R_Cerebellum 21		-41	-31
R_IPC 60		-34	27
R_Insula 46		-6	5
R_M1 6		-30	72
R_PAC 51		-13	4
R_PMd 48		6	51
R_PMv 54		5	6
R_Putamen 29		-4	10
R_S1 10		-37	72
R_S2 46		-21	13
R_SMA 4		-5	64
R_SPL 14		-43	77
R_Thalamus 18		-16	9
CN_L -	4.6	-31.1	-12.3
CN_R 5.	6	-31.1	-12.3
medulla_Raphe 0		-39.8	-46.9
pons_Raphe 0		-38.8	-35.5
pons_medulla_junction_L -	2.34	-39.5	-45.7
pons_medulla_junction_R 4.	12	-38.2	-41.2
PPN_L -	5.53	-30.4	-15.9
PPN_R 6.	53	-30.4	-15.9
RedN_L -	5.74	-19.7	-8.62
RedN_R 5.	74	-19.7	-8.62

References

[1] Napadow V, Dhond R, Kennedy D, Hui KK, Makris N. Automated brainstem co-registration (ABC) for MRI. Neuroimage. 2006. 32(3): 1113-9.

[2] Li X, Chen L, Kutten K, et al. Multi-atlas tool for automated segmentation of brain gray matter nuclei and quantification of their magnetic susceptibility. Neuroimage. 2019. 191: 337-349.

[3] Visser E, Keuken MC, Forstmann BU, Jenkinson M. Automated segmentation of the substantia nigra, subthalamic nucleus and red nucleus in 7T data at young and old age. Neuroimage. 2016. 139: 324-336.

[4] Tang Y, Sun W, Toga AW, Ringman JM, Shi Y. A probabilistic atlas of human brainstem pathways based on connectome imaging data. Neuroimage. 2018. 169: 227-239.

[5] Paxinos G, Huang XF. Atlas of the human brainstem. 1995. 1st. San Diego. ACADEMIC PRESS.

[6] Naidich TP, Duvernoy HM, Delman BN, Sorensen AG, Kollias SS, Haacke EM. Duvernoy's Atlas of the Human Brain Stem and Cerebellum. 2009. 1st. Austria. Springer-Verlag/Wien. 63-89.

[7] Petersen KJ, Reid JA, Chakravorti S, et al. Structural and functional connectivity of the nondecussating dentato-rubro-thalamic tract. Neuroimage. 2018. 176: 364-371.

[8] Beissner F, Deichmann R, Baudrexel S. fMRI of the brainstem using dual-echo EPI. Neuroimage. 2011. 55(4): 1593-9.
